# Independent associations of urinary albumin-to-creatinine ratio and serum cystatin C with carotid intima-media thickness in community-living Taiwanese adults

**DOI:** 10.1186/s12882-020-02123-x

**Published:** 2020-10-31

**Authors:** Chiu-Shong Liu, Chia-Ing Li, Yuh-Cherng Guo, Chih-Hsueh Lin, Wen-Yuan Lin, Chung-Hsiang Liu, Mu-Cyun Wang, Chuan-Wei Yang, Shing-Yu Yang, Tsai-Chung Li, Cheng-Chieh Lin

**Affiliations:** 1 School of Medicine, College of Medicine, China Medical University, Taichung, Taiwan; 2 Department of Family Medicine, China Medical University Hospital, Taichung, Taiwan; 3 Department of Medical Research, China Medical University Hospital, Taichung, Taiwan; 4 Department of Neurology, China Medical University Hospital, Taichung, Taiwan; 5 Department of Public Health, College of Public Health, China Medical University, 91 Hsueh-Shih Road, Taichung, 40421 Taiwan; 6 Department of Healthcare Administration, College of Medical and Health Science, Asia University, Taichung, Taiwan

**Keywords:** Intima-media thickness, Cystatin C, Creatinine, Albumin-to-creatinine ratio

## Abstract

**Background:**

Renal function is a key factor of cardiovascular disease. Carotid intima-media thickness (IMT) has been widely used as a marker of early subclinical atherosclerosis. The determinants of cystatin C, a novel marker of renal function, have not been extensively studied in the Asian population. This study aimed to assess the determinants of cystatin C and explore whether carotid thickening was associated with urinary albumin-creatinine ratio and cystatin C in community-living Taiwanese adults.

**Methods:**

A cross-sectional study was conducted on participants from Taichung City, Taiwan. All the participants underwent carotid ultrasonography. Carotid IMT-mean and IMT-maximum were derived. Kidney biomarkers were measured on the basis of urinary albumin-to-creatinine ratio (ACR) and cystatin C. Multiple linear regression analysis was used.

**Results:**

A total of 1032 individuals were recruited, and 469 (45.44%) of them were men. An increased cystatin C level was significantly associated with older age, male gender, lack of physical activity, low HDL cholesterol, abdominal obesity, high hs-CRP, and high ACR. The multivariate-adjusted mean carotid IMT-mean and IMT-maximum values significantly increased by 80.49 and 195.23 μm for every one unit of increase in cystatin C level and by 0.07 and 0.14 μm for every one unit of increase in ACR, respectively (all *p* < 0.001 except ACR on IMT-maximum with *p* < 0.01). Lack of physical activity, low HDL, abdominal obesity, high hs-CRP, and high ACR were the determinants of cystatin C.

**Conclusion:**

Cystatin C and ACR were strongly and linearly associated with carotid thickening, a marker of subclinical atherosclerosis.

**Supplementary Information:**

The online version contains supplementary material available at 10.1186/s12882-020-02123-x.

## Background

Cardiovascular disease (CVD) is one of the causes of morbidity and premature mortality and the main cause of death worldwide, including Taiwan. The development of metabolic syndrome, including the cluster of CVD risk factors, results from two determinants, namely, genetic influence and environments, which increases the risk of developing atherosclerosis. Renal function has been regarded as a risk factor of CVD [1, 2].

Cystatin C is considered as an ideal marker of renal function [3] and more precise than serum creatinine levels in detecting early kidney dysfunction [4, 5]. Plasma cystatin C measurement is superior to creatinine-based methods for estimating the glomerular filtration rate [3, 6] in terms of its greater sensitivity than creatinine as a predictor of cardiovascular risk and for the early diagnosis of early chronic renal disease. Cystatin C is linked to pathophysiological changes in the vascular wall [7, 8]. In addition to cystatin C, albuminuria is a renal marker associated with an increased risk of CVD. Albuminuria, a marker of kidney damage, is the consequence of albumin leakage across the glomerular podocyte filtration barrier into the urine and the increased excretion of urinary albumin. Albuminuria is also a marker of generalized endothelial dysfunction in which accelerated atherosclerosis [9] has been known as a sensitive prognostic marker to evaluate the increased risk of CVD [10, 11]. Urinary albumin-creatinine ratio (ACR) is a preferred approach for albuminuria detection.

Carotid intima-media thickness (IMT), the measurement of the intima and media layers of the artery, has been described as a quantitative measure of subclinical atherosclerosis [12]. A gradual increase in carotid IMT is characterized by changes in arterial wall vessels during atherosclerosis progression. Carotid IMT, which is used as a biomarker for the early clinical diagnosis of CVD, is one of the most commonly employed and well established for early surrogate biomarkers of atherosclerosis [13, 14] and has been reported to predict CVD and myocardial infarction [13, 15]. Recent studies on the association between cystatin C and carotid IMT have yielded inconsistent results [16–26]. Some prior research demonstrated that increased cystatin C is associated with carotid IMT [24]. On the contrary, no association between cystatin C and carotid IMT has been found in another study [22]. Among the investigations that confirmed an association between cystatin C and carotid IMT, only one study has been performed in the general population. The association between cystatin C and arteriosclerosis in adults residing in the community has been rarely discussed. Thus, this study evaluated whether independent cross-sectional associations existed between urinary ACR and cystatin C with subclinical atherosclerosis in participants of the Taichung Community Health Study (TCHS). This study also explored the determinants of cystatin C.

## Methods

### Study design and study subjects

A community-based cross-sectional study was conducted in 2015. All participants from three studies on TCHS, TCHS for elders (TCHS-E), and TCHS for family members (TCHS-F) were contacted individually via a phone call and a letter to invite them to participate in this study, including providing extracranial carotid artery ultrasound measurement and a fasting blood sample. The TCHS, TCHS-E, and TCHS-F studies are described in the Supplementary Information for the Study Subjects. In brief, TCHS was a population-based prospective cohort study conducted in 2004. The sampling design was a two-stage approach, with each stage having a sampling rate proportional to size. The target population was defined as residents aged 40 years and over in Taichung City, Taiwan, in 2004. A total of 2359 participants were involved, with an overall response rate of 66.83%. In the second wave, 1666 participants were included, with a follow-up rate of 70.62%. A total of 1136 participants returned, with an overall follow-up rate of 50.8% in the third wave (2010–2013). TCHS-E was also a population-based cohort study. The study population included residents aged 65 years and over in eight Lis (administrative neighborhoods) in North District in Taichung City, Taiwan, in 2009. A total of 2750 subjects were eligible, and 1347 agreed to participate, with an overall response rate of 49.0%. After 1 year, 1078 subjects were followed up, with a follow-up rate of 81.3%. TCHS-F was a family study that aimed to examine the familiar aggregation of cardiovascular risk factors. In this family study, 807 families with at least one first-degree relative of participants from TCHS or TCHS-E were recruited. The inclusion criteria of TCHS-F were individuals who were spouses or first-degree blood relatives (parents, full siblings, and offspring) aged 20 years or over of the participants of TCHS or TCHS-E. A total of 1919 family members of TCHS or TCHS-E participated. In the present cross-sectional study, the cystatin C levels of 1039 study subjects who were participants of TCHS, TCHS-E, and TCHS-F studies and had their attendance in August 2016–April 2018 were measured because of the budget limit. Seven subjects with missing values were excluded from the analysis. This study was approved by the Human Research Committee of China Medical University Hospital (CMUH105-REC1–002), all methods were carried out in accordance with relevant guidelines and regulation, and informed consent was obtained from each participant.

### Measurements

A standardized physical check-up procedure and a questionnaire were used to complete data collection. Sociodemographic factors included age and sex. Lifestyle behaviors included alcohol drinking, physical activity, and smoking. Disease histories of diabetes, hypertension, heart disease, hyperuricemia, hyperlipidemia, stroke, cancer, and medication history were obtained. Lifestyle behaviors and disease histories were categorized as “Yes” and “No” classes.

Anthropometric measurements and blood samples were obtained. Weight and height were measured using an autoanthropometer (super-view, HW-666). During measurement, the subjects were shoeless and wearing light clothing. Body mass index (BMI) was derived by dividing weight (kg) by (height)^2^ (m^2^). Waist circumference was measured as the midway point between the crest of the ilium and the inferior margin of the last rib in a horizontal plane while the participant was standing. WC was measured to the nearest 0.1 kg. Blood pressure was measured in a seated position by using an electronic device (OMRON, HEM-770A, Japan).

Blood was drawn in the morning after a 12-h overnight fasting with minimal trauma from an antecubital vein. Biochemical markers, including fasting plasma glucose, triglyceride, HDL (high density lipoprotein)-cholesterol, uric acid, urine albumin, and creatinine, were analyzed with a biochemical autoanalyzer (Beckman Coluter, Lx-20, USA) at the Clinical Laboratory Department of China Medical University Hospital. Fasting plasma glucose level was measured via a glucose oxidase method (Astra-8, Beckman, CA, USA). Urinary ACR was measured using the morning urine sample as a marker of the albumin excretion rate. Urinary ACR ranging from 30 mg g^− 1^ creatinine to 300 mg g^− 1^ creatinine was defined as microalbuminuria, while urinary ACR above 300 mg g^− 1^ was defined as macroalbuminuria. The hs-CRP (high sensitivity C-reactive protein) level was determined through nephelometry, a latex particle-enhanced immunoassay (TBA-200FR, Tokyo, Japan) with the inter- and intra-assay coefficient of variations of < 2.0 and 1.9%, respectively. The lower detection limit of the assay was 0.1 mg/L. Cystatin C was identified via a particle-enhanced immunonephelemetric assay (N Latex Cystatin C, Dade-Behring, Marburg, Germany) with a nephelometer (BNII, Dade Behring). The modified definition of metabolic syndrome as described in the Third Report of the National Cholesterol Education Program’s Adult Treatment Panel (ATP III) was used [27]. According to the ATP III, the metabolic syndrome components were as follows: hyperglycemia (fasting plasma glucose ≥110 mg/dl), hypertriglyceridemia (serum triglycerides ≥150 mg/dl), hypertension (blood pressure ≥ 130/85 mmHg), abdominal obesity (waist circumference > 90 cm in men and waist circumference > 80 cm in women), and low HDL cholesterol (serum HDL-cholesterol < 40 mg/dl in men and HDL-cholesterol < 50 mg/dl in women).

### Extracranial carotid artery ultrasound measurement

The IMT measurements of all the study subjects were measured with the same machine (GE L7000, GE, Milwaukee, Wis., USA) under the standardized procedure. After resting for about 10 min in the supine position with their necks in a slight hyperextension, all the study subjects underwent carotid ultrasound examination with a 7.5 MHz probe to bilaterally scan the far and near walls of arterial segments and obtain the transverse and longitudinal (posterior oblique, lateral, and anterior oblique) views. Each ultrasound image, including atherosclerotic plaques and the intima-media complex thicknesses, was recorded in an online digital filing system via a computer. The arterial segments were as follows: (1) the proximal (first 1 cm) of the internal carotid arteries (ICAs); (2) 1–2 cm proximal to the tip of the flow divider into the right common carotid arteries (CCAs); and (3) the carotid bifurcations (bulb) beginning at the tip of the flow divider and extending 1 cm proximal to the flow divider tip. The maximal IMT of each segment at the CCA was derived from a minimum of three frames as the maximum IMT (segment-specific IMT-maximum) and mean IMT (segment-specific IMT-mean) of each segment by averaging these frames. The total carotid IMT (IMT-mean or IMT-maximum) was calculated as a composite measure (mean or maximum of the eight carotid sites) that combined the near and far walls of CCA IMT, bulb IMT, and ICA IMT of both sides of the neck. IMT-maximum was expressed as the mean of the maximum values of the eight carotid sites, while the IMT-mean was defined as the mean of the average values of the eight carotid sites. The IMT measurements were operated by two operators. The intra-operators reliability of IMT-mean for the first and second operators were 0.85 and 0.89, respectively; the intra-operators reliability of IMT-maximum were 0.85 and 0.97, respectively; and the inter-operators reliability of IMT-mean and IMT-maximum were both 0.88.

### Statistical analysis

Simple descriptive analyses, such as proportion, mean, and standard deviation, were applied to analyze data when appropriate. The differences between cystatin C and ACR groups were evaluated through analysis of variance. For categorical variables, comparisons were assessed via a Chi-square or Fisher’s exact test. Multiple linear regression models were used on the basis of cross-sectional data to investigate the determinants of cystatin C and whether cystatin C and ACR were associated with carotid IMT after the metabolic syndrome components, inflammation markers, and lifestyle behaviors that were significantly associated with carotid IMT were considered. In order to estimate the independent association between cystatin C or ACR with carotid IMT, cystatin C and ACR were entered into the multivariate models simultaneously. All analyses were conducted with SAS version 9.4 (SAS, Cary, NC). *P* values were two tailed, and significance level was set at 0.05.

## Results

A total of 1032 subjects were analyzed between August 2016 and December 2018. About half of the participants were female (55%). The mean ages of men and women were 69.80 years (standard deviation = 9.71 years) and 66.07 years (9.44 years), respectively. The characteristics of the study subjects across the tertiles of cystatin C and albuminuria status are presented in Table 1. The highest tertile of cystain C was associated with the high proportions of men, smoking, lack of physical activity, hyperglycemia, hypertriglyceridemia, hypertension, low HDL cholesterol, and high mean values of age, fasting insulin, hs-CRP, carotid IMT-mean, and IMT-maximum. The results of the comparison of the characteristics of the three albuminuria groups (normal albuminuria [< 30 mg/g], microalbuminuria [30–300 mg/g], and macroalbuminuria [≥300 mg/g]) are shown in Table 1. The values of carotid IMT-mean and IMT-maximum in the normal group were significantly lower than those in the microalbuminuria and macroabluminuria groups.

**Table 1:** Characteristics of study subjects according to cystatin C and albumin-to-creatinine ratio

	Cystatin C				ACR			
Variables	≤0.87 (*N* = 358)	0.88–1.01 (*N* = 327)	> 1.02 (*N* = 347)	*P* value	Normal (*N* = 888)	Microalbuminuria (*N* = 119)	Macroalbuminuria (*N* = 25)	*P* value
***Socio-demographic factors***								
Age (years)	63.88 ± 8.17	66.65 ± 5.74	72.83 ± 9.69	< 0.001	66.78 ± 9.36	73.81 ± 9.92	74.19 ± 9.53	< 0.001
Sex				< 0.001				< 0.001
Men	100 (27.93)	151 (46.18)	218 (62.82)		377 (42.45)	77 (64.71)	15 (60.00)	
Women	258 (72.07)	176 (53.82)	129 (37.18)		511 (57.55)	42 (35.29)	10 (40.00)	
***Lifestyle behaviors***								
Smoking	4 (1.12)	23 (7.03)	21 (6.05)	< 0.001	38 (4.28)	8 (6.72)	2 (8.00)	0.36
Alcohol drinking	25 (6.98)	37 (11.31)	34 (9.8)	0.14	78 (8.78)	17 (14.29)	1 (4.00)	0.10
Physical activity	291 (81.28)	264 (80.73)	255 (73.49)	0.02	704 (79.28)	90 (75.63)	16 (64.00)	0.13
***Metabolic Syndrome components***								
Hyperglycemia	110 (30.73)	107 (32.72)	156 (44.96)	< 0.001	295 (33.22)	63 (52.94)	15 (60.00)	< 0.001
Hypertriglyceridemia	96 (26.82)	104 (31.8)	126 (36.31)	0.03	265 (29.84)	49 (41.18)	12 (48.00)	0.009
Hypertension	200 (55.87)	213 (65.14)	269 (77.52)	< 0.001	554 (62.39)	107 (89.92)	21 (84.00)	< 0.001
Abdominal obesity	256 (71.51)	227 (69.42)	214 (61.67)	0.01	599 (67.45)	77 (64.71)	21 (84.00)	0.17
Low HDL cholesterol	116 (32.4)	133 (40.67)	158 (45.53)	0.002	335 (37.73)	55 (46.22)	17 (68.00)	0.003
***Biochemical lab parameters***								
Fasting insulin (IU/L)	5.21 (3.26)	5.74 (3.49)	7.81 (12.52)	< 0.001	5.72 (3.51)	7.85 (9.87)	17.62 (39.34)	< 0.001
hs-CRP (mg/L)	0.13 (0.25)	0.15 (0.26)	0.27 (0.81)	< 0.001	0.17 (0.48)	0.28 (0.74)	0.20 (0.35)	0.10
***IMT measurements***								
IMT-Mean (μm)	1417.65 ± 531.28	1570.82 ± 644.37	1947.09 ± 821.41	< 0.001	1576.36 ± 671.91	2050.36 ± 809.16	2120.52 ± 776.11	< 0.001
IMT-Maximum (μm)	926.32 ± 196.75	999.17 ± 274.33	1138.98 ± 334.66	< 0.001	993.08 ± 268.27	1176.91 ± 339.45	1266.72 ± 339.83	< 0.001

Differences in continuous variables were tested using student t test. Differences in categorical variables were tested using the Chi-square test

*HDL* High-density lipoprotein, *hs-CRP* high sensitivity C-reactive protein, *IMT* intima-media thickness, *ACR* albumin-to-creatinine ration

Table 2 presents the multiple linear regression models used to explore the determinants of cystatin C. When age and gender were entered into the model, 12.2% of the variation in cystatin C was explained, and both age and gender were significantly associated with cystatin C. When the lifestyle behaviors of alcohol drinking, smoking, and physical activity were further considered, physical activity was the only significant variable. The effect of age and gender remained similar and explained 1.1% of additional variation in cystatin C. When metabolic syndrome components were entered into the model, abdominal obesity and low HDL cholesterol were the two significant variables, and the effect of physical activity was attenuated but was still significant. When the biochemical lab parameters were included, hs-CRP and ACR were the significant variables that were associated with cystatin C, and the variation in cystatin C improved from 15.0 to 34.3%.

**Table 2:** Multivariate analysis of sociodemographic characteristics, lifestyle behaviors, metabolic syndrome components, and laboratory features on serum cystain C level

Variables	Cystatin C (mg/L) β (SE)			
	Model 1	Model 2	Model 3	Model 4
***Socio-demographic factors***				
Age (years)	0.01 (0.001)***	0.01 (0.001)***	0.01 (0.001)***	0.01 (0.001)***
Men	0.10 (0.02)***	0.10 (0.03)***	0.12 (0.03)***	0.11 (0.02)***
***Lifestyle behaviors***				
Smoking		0.02 (0.06)	0.02 (0.06)	0.03 (0.05)
Alcohol drinking		−0.04 (0.04)	−0.04 (0.04)	−0.03 (0.04)
Physical activity		−0.10 (0.03)***	−0.08 (0.03)**	−0.06 (0.03)*
***Metabolic Syndrome components***				
Hyperglycemia			−0.003 (0.03)	− 0.01 (0.02)
Hypertriglyceridemia			−0.04 (0.03)	−0.02 (0.03)
Hypertension			−0.001 (0.03)	−0.01 (0.02)
Abdominal obesity			0.07 (0.03)**	0.05 (0.02)*
Low HDL cholesterol			0.10 (0.03)***	0.07 (0.03)*
***Biochemical lab parameters***				
hs-CRP (mg/L)				0.06 (0.02)**
ACR (mg/g)				0.001 (0.00002)***
***R***^***2***^	12.2%	13.3%	15.0%	34.3%

*SE* standard error, *HDL* high-density lipoprotein, *hs-CRP* high sensitivity C-reactive protein, *ACR* albumin-to-creatinine ration

*: *p* < 0.05; **: *p* < 0.01; ***: *p* < 0.001

Table 3 shows the adjusted means of IMT-mean IMT-maximum across various cystatin C levels and albuminuria status. After adjustments for age and sex were made, the mean carotid IMT-mean and IMT-maximum were significantly different in cystatin C levels and albuminuria status. After the lifestyle behaviors of smoking, alcohol drinking, and physical activity were further considered, the results remained similar. After further adjustments for metabolic syndrome components, fasting insulin, and hs-CRP were made, the differences in the IMT-mean of the cystatin C levels became borderline significant and remained significant across albuminuria categories. The following adjusted mean values (95% confidence interval) were obtained on the basis of the tertiles of cystatin C: for the carotid IMT-mean, 1069.09 (1021.26, 1116.92), 1075.83 (1030.80, 1120.87), and 1112.06 (1066.09, 1158.04); for IMT-maximum, 1790.81 (1670.17, 1911.46), 1797.65 (1684.04, 1911.25), and 1937.21 (1821.24, 2053.18); for the IMT-mean of the ACR groups (normal albuminuria, microalbuminuria, and macroalbuminuria), 1077.27 (1036.05, 1118.50), 1123.70 (1066.55, 1180.84), and 1201.22 (1097.91, 1304.52); and for the IMT-maximum, 1819.39 (1715.07, 1923.70), 1974.65 (1830.06, 2119.24), and 2013.4 (1752.02, 2274.77).

**Table 3:** Carotid intima-media thickness values by cystatin C and albumin-to-creatinine ratio

Variables	IMT-mean (95% CI)			IMT-Maximum (95% CI)		
	Age- and sex adjusted	Multivariate adjusted^1^	Multivariate adjusted^2^	Age- and sex adjusted	Multivariate adjusted^1^	Multivariate adjusted^2^
***Cystatin C (mg/L)***						
≤ 0.87^(1)^	997.23 (970.72, 1023.75)	1062.43 (1015.31, 1109.56)	1069.09 (1021.26, 1116.92)	1569.78 (1502.88, 1636.68)	1769.61 (1650.97, 1888.25)	1790.81 (1670.17, 1911.46)
0.88–1.01^(2)^	1017.43 (991.22, 1043.64)	1076.29 (1032.18, 1120.39)	1075.83 (1030.8, 1120.87)	1611.26 (1545.12, 1677.39)	1793.05 (1682.01, 1904.08)	1797.65 (1684.04, 1911.25)
> 1.02^(3)^	1060.18 (1033.30, 1087.05)	1120.55 (1075.41, 1165.69)	1112.06 (1066.09, 1158.04)	1770.06 (1702.25, 1837.88)	1951.67 (1838.02, 2065.31)	1937.21 (1821.24, 2053.18)
*p* value	0.007	0.01	0.08	< 0.001	< 0.001	0.006
Tukey test	3 > 1	3 > 1		3 > 1,2	3 > 1,2	3 > 1,2
***ACR groups***						
Normal ^(1)^	1013.48 (997.47, 1029.49)	1075.09 (1034.81, 1115.38)	1077.27 (1036.05, 1118.50)	1620.48 (1579.92, 1661.03)	1808.75 (1706.94, 1910.57)	1819.39 (1715.07, 1923.70)
Microalbuminuria ^(2)^	1081.06 (1036.77, 1125.34)	1139.73 (1083.53, 1195.93)	1123.70 (1066.55, 1180.84)	1829.33 (1717.15, 1941.51)	2005.57 (1863.53, 2147.61)	1974.65 (1830.06, 2119.24)
Macroalbuminuria ^(3)^	1169.92 (1075.19, 1264.66)	1227.62 (1126.64, 1328.6)	1201.22 (1097.91, 1304.52)	1894.1 (1654.1, 2134.1)	2064.19 (1808.97, 2319.42)	2013.40 (1752.02, 2274.77)
*p* value	< 0.001	< 0.001	0..01	< 0.001	0.001	0.01
Tukey test	2,3 > 1	2,3 > 1	3 > 1	2 > 1	2 > 1	2 > 1

Multivariate adjusted^1^ age, sex, smoking, alcohol drinking, and physical activity

Multivariate adjusted^2^ age, sex, smoking, alcohol drinking, physical activity, metabolic syndrome components, fasting insulin and hs-CRP

*IMT* intima-media thickness, *CI* confidence interval, *ACR* albumin-to-creatinine ration

*: *p* < 0.05; **: *p* < 0.01; ***: *p* < 0.001

Table 4 lists the estimated differences in the carotid IMT-mean and IMT-maximum. The carotid IMT-mean and IMT-maximum values from the first to the third tertile groups of cystatin C and from the normal to the macroalbuminuria group increased. The multivariate-adjusted mean carotid IMT-mean and IMT-maximum values significantly increased by 80.49 and 195.23 μm for every one unit increase in cystatin C, respectively, and by 0.07 and 0.14 μm for every one unit increase in ACR (all *p* < 0.001 except ACR on IMT-maximum with *p* < 0.01), respectively.

**Table 4:** Multivariate analysis of carotid intima-media thickness for baseline characteristics, lifestyle behaviors, disease histories, serum cystain C and albumin-to-creatinine ration

Variables	IMT mean β (SE)			IMT-Maximum β (SE)		
	Age- and sex adjusted	Multivariate adjusted^1^	Multivariate adjusted^2^	Age- and sex adjusted	Multivariate adjusted^1^	Multivariate adjusted^2^
***Cystatin C (mg/L)***						
≤ 0.87	ref	ref	ref	ref	ref	ref
0.88–1.01	18.42 (18.66)	12.36 (18.67)	6.42 (18.63)	36.89 (47.12)	19.63 (47.07)	5.44 (47.03)
> 1.02	52.60 (20.29)**	48.59 (20.35)*	37.89 (20.54)	177.35 (51.24)***	161.35 (51.29)**	134.76 (51.86)**
Continuous β (SE), per unit	88.71 (19.53)***	87.74 (19.56)***	80.49 (19.67)***	219.58 (49.46)***	210.80 (49.45)***	195.23 (49.76)***
***ACR groups***						
Normal	ref	ref	ref	ref	ref	ref
Microalbuminuria	61.06 (24.29)*	58.66 (24.22)*	42.78 (24.37)	185.85 (61.34)**	176.06 (61.06)**	141.62 (61.50)*
Macroalbuminuria	141.86 (49.28)**	139.41 (49.19)**	116.47 (50.24)*	220.74 (124.47)	208.90 (123.99)	165.18 (126.81)
Continuous β (SE), per unit	0.08 (0.02)***	0.08 (0.02)***	0.07 (0.02)***	0.15 (0.05)**	0.15 (0.05)**	0.14 (0.05)**

Multivariate adjusted^1^ age, sex, smoking, alcohol drinking, and physical activity

Multivariate adjusted^2^ age, sex, smoking, alcohol drinking, physical activity, metabolic syndrome components, fasting insulin and hs-CRP

*IMT* intima-media thickness, *SE* standard error, *ACR* albumin-to-creatinine ration

*: *p* < 0.05; **: *p* < 0.01; ***: *p* < 0.001

## Discussion

In this cross-sectional study performed with 1032 community-dwelling adults, older age, male gender, lack of physical activity, low HDL cholesterol, abdominal obesity, high hs-CRP, and high ACR were associated with increased cystatin C levels. High cystatin C and ACR levels were independently associated with an increase in carotid IMT-mean and IMT-maximum measurements.

Our findings showed that the metabolic components of abdominal obesity and low HDL cholesterol were determinants of cystatin C, and they were consistent with those of prior studies showing that cystatin C is associated with metabolic syndrome [28, 29], obesity determined by BMI [30–32], and HDL [33]. A cross-sectional study on 925 patients with dyslipidemia and 26% prevalence of metabolic syndrome has revealed that cystatin C is associated with metabolic syndrome in these patients [28]. The Chennai Urban Rural Epidemiology Study, a population-based study in southern India, included 40 subjects with four or five metabolic components, 40 subjects with three metabolic components, 37 subjects with two metabolic components, 44 subjects with one metabolic component, and 43 subjects without a metabolic component. Their findings indicated that cystatin C is linearly associated with the number of metabolic components [28, 29]. In a national cross-sectional study from the Third National Health and Nutrition Examination Survey on 5083 participants with a normal renal function defined by an estimated glomerular filtration rate of ≥60 mL/min/1.73 m^2^ without albuminuria, the association between BMI and increased serum cystatin C levels is explored, revealing a graded relationship between higher BMI and increased serum cystatin C [30]. A cross-sectional study on 2583 participants without clinically recognized chronic kidney disease from the National Health and Nutrition Examination Survey 1999 to 2002 identified the association between obesity determined by BMI and cystatin C independent of age, sex, race, education, smoking, alcohol intake, cholesterol, and CRP levels [32]. In a cross-sectional study on 159 community-living elderly Japanese women, HDL is negatively linked with cystatin C after adjustments for creatinine-based estimated glomerular filtration rate are made [33]. According to the definition of metabolic syndrome by the Third Report of the National Cholesterol Education Program’s Adult Treatment Panel [27], abdominal obesity should be determined by waist circumference. However, most prior studies adopted obesity as determined by BMI by estimating the overall body fat mass. Our study demonstrated that abdominal obesity, as determined by waist circumference, was correlated with cystatin C.

The pathogenesis of CVD appears to be multifactorial, involving genetic and acquired factors that play a key role in the pathway of inflammation that leads to CVD. Inflammation is crucial in various endothelial function aspects, which are critical to an early stage of atherosclerosis and its associated clinical risk factors [34]. Our study found that cystatin C was linked with hs-CRP, thereby suggesting that cystatin C was involved in the pathway of the potential pathogenesis of CVD; this result can be supported by previous findings, which indicated that cystatin C is a relevant marker of the risk of CVD among relatively healthy middle-aged adults [35], predicts incident type 2 diabetes [36] and progression from normal fasting plasma glucose to prediabetes [37], and predicts the cardiovascular risk in elderly persons [38, 39] and adults of the general population [40].

Increased cystatin C levels are associated with carotid IMT in a multi-ethnic study on atherosclerosis [24], in the general population [23], in the health check-up population [20], in postmenopausal women [21], in metabolic syndrome patients [19], in patients with hypertension [16, 17, 25], and in patients with type 2 diabetes [26, 41]. By contrast, a study investigated 446 Japanese adults without chronic kidney disease and proteinuria and did not find the relationship of cystatin C and carotid IMT [22]. In a study on a healthy Chinese population with a normal renal function and without cardiovascular disease, cystatin C is associated with carotid IMT, but this association is explained when BMI, systolic and diastolic blood pressure, lipid profiles, albumin, fasting blood glucose, and inflammatory markers are considered [18]. A study on African descent has revealed that cystatin C is not associated with carotid IMT beyond traditional cardiovascular risk factors [42]. The association between cystatin C and arteriosclerosis in middle-aged adults has been less consistent. Thus, we measured cystatin C and investigated its association with carotid IMT in TCHS. Our study supported previous results, which indicated that cystatin C can be used as a precise indicator to help the early diagnosis of atherosclerosis in community-based adults.

Our study found that ACR can explain the variation in cystatin C in this community-based sample, but both are independently associated with carotid IMT. Prior studies explored the associations between renal biomarkers and carotid IMT in the general population by focusing on the measures of the blood urea nitrogen [24] and creatinine-based estimated glomerular filtration rate [19, 24]. Our study reported an association between ACR and carotid IMT values in the participants of the general population, and this result was consistent with previous findings in patients with type 2 diabetes [43, 44].

In order to evaluate whether serum cystatin C is more important than creatinine or creatinine-based estimated glomerular filtration rate (eGFR) in term of capturing their relationship with IMT, we performed additional analysis by adding the eGFR and creatinine separately into the multivariate models with cystatin C and ACR (Supplemental Tables 1 and 2). The reason why we cannot enter eGFR and serum creatinine into the multivariate models at the same time is because eGFR is derived from serum creatinine and they shared too much common information. We found cystatin C was significantly associated with IMT-maximum when eGFR or creatinine was considered while either eGFR or creatinine was not associated with IMT-maximum. Cystatin C was associated with IMT-mean at borderline significant when creatinine was considered but cystatin C was not associated with IMT-mean when eGFR was taken into account. Our findings support prior studies that cystatin C may be a better measurement than creatinine-based methods in terms of capturing its relation to carotid IMT.

The strengths of this study included a well-defined sample of adults from the general population in Taiwan by using standard questionnaires and instruments. A complete physical check-up was taken by all the participants. Vigorous quality assurance programs and a strict personnel training process were set up. Thus, the percentage of missing data was low (< 0.5%). Our study also had standard laboratory approaches for glucose and lipid measurements within a central clinical lab for the same assays.

This study also had three notable limitations. First, the use of a cross-sectional study design would be relevant to the interpretation of our results; thus, we could not infer the causal pathways underlying the observed relationships. Second, the study was restricted to the first 1032 participants, and a potential selection bias might exist. The demographic characteristics of the individuals with and without cystatin C measurement were assessed by comparing age and gender for the possibility of selection bias, and similar distributions were found. Given that age and gender distributions did not differ, this kind of selection error might be random. Thus, the resulting bias in the effect might be negligible, and it could pose a low threat to the internal validity of our study findings. Lastly, our study was based on a Taiwanese population with main Han Chinese. Such a selection bias may limit our findings to be generalized to other ethnic populations, but they may be generalizable to other Chinese populations having similar characteristics as our study sample.

## Conclusions

In a cross-sectional study on 1032 community-living Taiwanese adults, older age, male gender, lack of physical activity, low HDL cholesterol, abdominal obesity, high hs-CRP, and high ACR were independently linked with a high cystatin C level. Increased serum cystatin C and ACR were linearly associated with carotid IMT measurements. The early detection of subclinical atherosclerosis might target those with increased cystatin C and ACR levels. Further research is warranted to assess whether reducing the risk factors of increased serum cystatin C favorably affected serum cystatin C levels and subclinical atherosclerosis development.

## Supplementary Information

**Additional file 1: Figure S1.** Time points of the data collection for the Taichung Community Health Study (TCHS), Taichung Community Health Study-Elder (TCHS-E), and Family Study of TCHS and TCHS-E. **Table S1.** Multivariate analysis of carotid intima-media thickness for serum cystain C, albumin-to-creatinine ration, and estimated glomerular filtration rate along with traditional risk factors. **Table S2.** Multivariate analysis of carotid intima-media thickness for serum cystain C, albumin-to-creatinine ration, and serum creatinine along with traditional risk factors.

## Data Availability

The dataset analyzed during the current study are available from the corresponding author on reasonable request.

## Abbreviations

CVD: Cardiovascular disease
ACR: Albumin-creatinine ratio
IMT: Intima-media thickness
TCHS: Taichung Community Health Study
TCHS-E: Taichung Community Health Study for elders
TCHS-: Taichung Community Health Study for family members
BMI: Body mass index
HDL: High density lipoprotein
hs-CRP: High sensitivity C-reactive protein
ATP III: Third Report of the National Cholesterol Education Program’s Adult Treatment Panel
ICA: Internal carotid arteries
CCAs: Common carotid arteries
Bulb: Bifurcations
eGFR: Estimated glomerular filtration rate
