# Supplementary material for: Independent associations of urinary albumin-to-creatinine ratio and serum cystatin C with carotid intima-media thickness in community-living Taiwanese adults
Source: BMC Nephrol. 2020 Oct 31;21:454. doi: 10.1186/s12882-020-02123-x (PMC7603773; doi:10.1186/s12882-020-02123-x)
Supplement: Supplementary file 1 — Additional file 1: Figure S1. Time points of the data collection for the Taichung Community Health Study (TCHS), Taichung Community Health Study-Elder (TCHS-E), and Family Study of TCHS and TCHS-E. Table S1. Multivariate analysis of carotid intima-media thickness for serum cystain C, albumin-to-creatinine ration, and estimated glomerular filtration rate along with traditional risk factors. Table S2. Multivariate analysis of carotid intima-media thickness for serum cystain C, albumin-to-creatinine ration, and serum creatinine along with traditional risk factors. [file 12882_2020_2123_MOESM1_ESM.docx]

**Supplementary information of the study subjects:**

TCHS, TCHS-E, and TCHS-F studies were briefly described as follows (Figure 2). A population-based cross-sectional study of TCHS was conducted in 2004. Residents aged 40 years and above and living in Taichung City, Taiwan, in 2004 were our target population. The sampling frame was the set of all family records from the Bureau of Households. A two-stage sampling design with a sampling rate proportional to size within each stage was used. At the first stage, the sampling unit was the Li (blocks of household units) with a selection probability of 0.125. A total of 39 Lis were selected from eight city districts. Then, 4,280 individuals were selected from the second stage. A total of 750 individuals who were ineligible were identified and excluded during household visits. Among 3,530 eligible subjects, 2,359 agreed to participate, and they had an overall response rate of 66.83%. This cohort was also longitudinally followed up from 2006 to 2018. A total of 1,666 residents participated in the second wave of data collection, with an overall loss to the follow-up rate of 29.38%. In the third wave of data collection (2010–2013), 1,136 participants returned for an overall follow-up rate of 50.8% after we excluded the participants who died during follow-up period. This study was approved by the Human Research Committee of China Medical University Hospital (DMR92-IRB-144, DMR93-IRB-138, DMR95-IRB-221, DMR 97-IRB-055 and DMR98-323). Informed consent was obtained from each participant.

TCHS-E was also a population-based cohort study. The target population consisted of all residents aged 65 years and above in eight administrative neighborhoods in the North District in Taichung City, Taiwan, in 2009. These eight administrative neighborhoods were selected for two reasons. First, they were administrative neighborhoods around our hospital, thereby facilitating future follow-up. Second, all districts in Taichung City have the same urbanization level. The age and gender distributions of these eight administrative neighborhoods are similar to those of Taichung and Taiwan populations. A total of 3,997 elderly residents were present during the time of the study. The sampling frame was the set of all individuals’ records from the Bureau of Households. During our household visits, 1,274 individuals who were ineligible were identified and excluded from the study sample. A total of 2,750 subjects were eligible, and 1,347 agreed to participate, with an overall response rate of 49.0%. After 1 year, 1,078 subjects were followed up, with a follow-up rate of 81.3%. This study was approved by the Human Research Committee of China Medical University Hospital (DMR 97-IRB-055). Written informed consent was obtained from each participant.

A family study on TCHS-F was conducted in 2010 to examine the familiar aggregation of cardiovascular risk factors. This family study recruited 807 families with at least one first degree relative of the participants from TCHS or TCHS-E. The inclusion criteria of TCHS-F were individuals who were spouses or first-degree blood relatives (parents, full siblings, and offspring) aged 20 years or above of the participants of TCHS or TCHS-E. A total of 1,919 family members of TCHS or TCHS-E participated. This study was approved by the Human Research Committee of China Medical University Hospital (DMR98-IRB-323). Written informed consent was obtained from each participant.


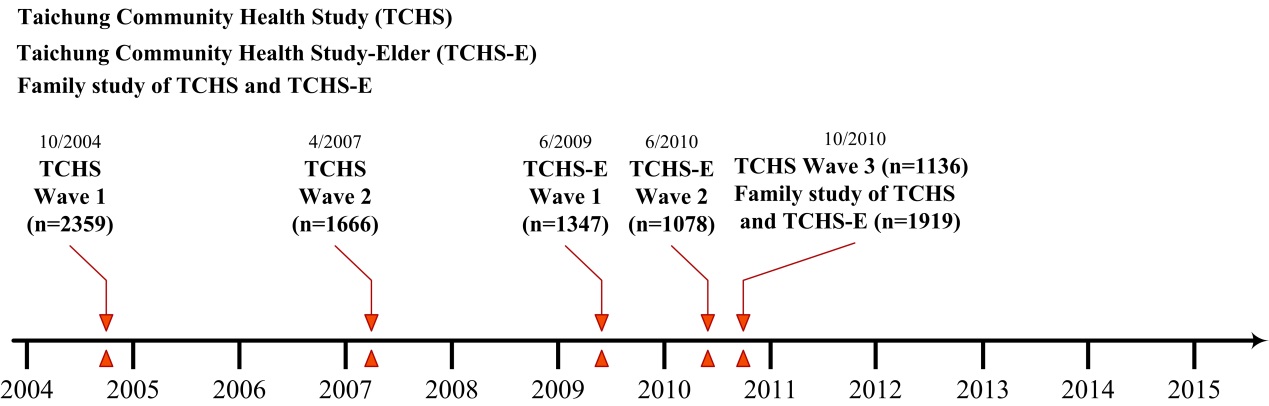


Supplement Figure 1. Time points of the data collection for the Taichung Community Health Study (TCHS), Taichung Community Health Study-Elder (TCHS-E), and Family Study of TCHS and TCHS-E.

Supplement Table 1. Multivariate analysis of carotid intima-media thickness for serum cystain C, albumin-to-creatinine ration, and estimated glomerular filtration rate along with traditional risk factors

| Variables | IMT-mean β (SE) | IMT-Maximum β (SE) |
| --- | --- | --- |
| ***Cystatin C (mg/L)*** |  |  |
| ≤0.87 | ref | ref |
| 0.88-1.01 | 8.65 (19.07) | 7.81 (48.16) |
| >1.02 | 30.75 (22.26) | 115.99 (56.22)* |
| ***ACR groups*** |  |  |
| Normal | ref | ref |
| Microalbuminuria | 38.68 (24.47) | 133.53 (61.80)* |
| Macroalbuminuria | 97.11 (51.48)^a^ | 124.73 (130.01) |
| ***eGFR (mL/min/1.73m^2^)*** |  |  |
| ≥90 | ref | ref |
| 89-60 | -6.33 (19.96) | -1.98 (50.40) |
| <60 | 42.40 (35.08) | 100.45 (88.59) |

IMT: intima-media thickness; SE: standard error; ACR: albumin-to-creatinine ration; eGFR: estimated glomerular filtration rate; *:p<0.05; **:p<0.01; ***:p<0.001; a: p=0.06.

Multivariate adjusted age, sex, smoking, alcohol drinking, physical activity, metabolic syndrome components, fasting insulin and hs-CRP.

Supplement Table 2. Multivariate analysis of carotid intima-media thickness for serum cystain C, albumin-to-creatinine ration, and serum creatinine along with traditional risk factors

| Variables | IMT-mean β (SE) | IMT-Maximum β (SE) |
| --- | --- | --- |
| ***Cystatin C (mg/L)*** |  |  |
| ≤0.87 | ref | ref |
| 0.88-1.01 | 7.33 (18.65) | 4.87 (47.11) |
| >1.02 | 39.16 (21.55) ^a^ | 130.13 (54.42)* |
| ***ACR groups*** |  |  |
| Normal | ref | ref |
| Microalbuminuria | 36.89 (24.89) | 144.28 (62.86)* |
| Macroalbuminuria | 96.68 (53.98) ^a^ | 169.01 (136.33) |
| ***Creatinine (mg/dL)*** |  |  |
| <1.0 | ref | ref |
| 1.00-1.49 | -13.19 (22.39) | 18.91 (56.56) |
| ≥1.5 | 41.67 (49.34) | -0.15 (124.62) |

IMT: intima-media thickness; SE: standard error; ACR: albumin-to-creatinine ration; *:p<0.05; **:p<0.01; ***:p<0.001; a: p=0.07.

Multivariate adjusted age, sex, smoking, alcohol drinking, physical activity, metabolic syndrome components, fasting insulin and hs-CRP.
